# High-gain observer-based nonlinear control scheme for biomechanical sit to stand movement in the presence of sensory feedback delays

**DOI:** 10.1371/journal.pone.0256049

**Published:** 2021-08-12

**Authors:** Nadia Sultan, Asif Mahmood Mughal, Muhammad Najam ul Islam, Fahad Mumtaz Malik

**Affiliations:** 1 Department of Electrical Engineering, Bahria University Islamabad, Islamabad, Pakistan; 2 Department of Electrical Engineering, CE&ME National University of Sciences and Technology Islamabad, Islamabad, Pakistan; Nankai University, CHINA

## Abstract

Sit-to-stand movement (STS) is a mundane activity, controlled by the central-nervous-system (CNS) via a complex neurophysiological mechanism that involves coordination of limbs for successful execution. Detailed analysis and accurate simulations of STS task have significant importance in clinical intervention, rehabilitation process, and better design for assistive devices. The CNS controls STS motion by taking inputs from proprioceptors. These input signals suffer delay in transmission to CNS making movement control and coordination more complex which may lead to larger body exertion or instability. This paper deals with the problem of STS movement execution in the presence of proprioceptive feedback delays in joint position and velocity. We present a high-gain observer (HGO) based feedback linearization control technique to mimic the CNS in controlling the STS transfer. The HGO estimates immeasurable delayed states to generate input signals for feedback. The feedback linearization output control law generates the passive torques at joints to execute the STS movement. The *H*_2_ dynamic controller calculates the optimal linear gains by using physiological variables. The whole scheme is simulated in MATLAB/Simulink. The simulations illustrate physiologically improved results. The ankle, knee, and hip joint position profiles show a high correlation of 0.91, 0.97, 0.80 with the experimentally generated reference profiles. The faster observer dynamics and global boundness of controller result in compensation of delays. The low error and high correlation of simulation results demonstrate (1) the reliability and effectiveness of the proposed scheme for customization of human models and (2) highlight the fact that for detailed analysis and accurate simulations of STS movement the modeling scheme must consider nonlinearities of the system.

## 1. Introduction

Human beings perform various tasks during their daily life which include walking, running, biking, jumping, mounting stairs, and sit-to-stand (STS). Among these tasks performing STS is one of the most complex and repetitive tasks that an individual performs in everyday life [1]. The proper execution of this task needs an adequate degree of mobility and thus enables an individual to maneuver independently [2]. It is the task that needs balance, coordination among different segments of a body, and ample strength in the lower limb to achieve successful execution [3]. STS movement due to its complex nature is not yet fully understood [4]. The dynamics of STS in humans need to be investigated and developed. In the context of subject research, designing a physiologically driven computational model of neural controller with good performance is a key element for many potential applications, for example, better prosthetic design and control, improve diagnosis and clinical interventions, the prospects of restoring mobility and rehabilitation processes, and improvement in design for assistive device. The control theory has a long history of providing insight into human motion and neural physiology. The control theoretical framework helps in understanding the posture and motion regulation mechanism of STS in the human neuromuscular system [5–7]. Several researchers have investigated motor regulation and motor learning in humans using mathematical models of musculoskeletal systems [8–12]. To narrow the focus, this paper presents a comprehensive mathematical framework for exploring posture and movement regulation mechanisms in the human neuromuscular control apparatus during STS transfer. To accomplish this, it is important to apply the knowledge of human neuromusculoskeletal motion control to build a physiology-based neural controller. A control structure is used to describe the decision-making role of the central nervous system (CNS). The modeling simulation scheme presented in this study provides insights that help us comprehend the human physiological system during STS movement. Gains at various joints indicate which joints require more effort during STS movement. This scheme illustrates the correlations between different kinematic variables and their impact on voluntary movement optimization.

Several studies have reported that the CNS regulates human motion and maintains balance by taking inputs from proprioceptors (Muscle Spindles (MS) and Golgi Tendon Organs (GTO)), tactile/somatosensory, visual, and vestibular systems [13–15]. Muscle feedback signals through proprioceptors suffer a delay in transmission to CNS [16,17]. These feedback delays are part of the physiological mechanism of humans and may differ in individuals depending on their health conditions and age [18]. Elderly subjects respond to external perturbations with longer latencies than young adults. This is due to altered or missing visual, vestibular, and proprioceptive information leading to instability or larger body exertion [19,20]. Wiesmeier et al. [18] used a model-based approach to identify the factors that mainly determine age-related changes in motor function. They collected the kinematic data from 20 healthy elderly subjects (mean age = 74 years) and compared it with the data of 19 healthy young subjects (mean age = 28 years) and 16 healthy middle-aged (mean age = 48 years). Based on findings they reported that elderly subjects prefer proprioceptive information over visual and somatosensory. Besides, they observed a decrease in the feedback amplitude and an increase in overall time delay, thus challenging the feedback system stability. These factors influence the joint torque, position, and velocity profiles and their optimum values during STS movement [21]. The joint torque is important to measure muscle activity in human performance. It also determines the practical loading conditions for prostheses including hip and knee replacement devices [22]. Thus, it is of great importance to consider the proprioceptive feedback delays in the model for evaluating the STS movement. Several model-based studies are conducted by researchers to study postural stability and STS movement. These studies are based on inverted pendulum body models. Iqbal [5] studied a four-segment biomechanical model for postural stability with proportional-integral-derivative (PID) controller for each degree-of-freedom (DOF). The PID was presumed to represent the CNS analog in the modeling paradigm. The results reported showed that the anatomical arrangement, muscle rigidity, force feedback, and physiological latencies play a major role in determining human motor control processes. This study was further extended by Ghulam Rasool et al. [21] to analyze the STS movement in the presence of physiological latencies. The authors simulated the model with TSK fuzzy modeling combined with the *H*_2_ control technique. They linearized the model at sitting and standing position and approximated delays with Pade approximation technique which increased the system order from 6^th^ order nonlinear to two 18^th^ order linear models. However, due to linearization at two points, the modeling scheme showed a larger deviation in angular profiles. The ankle, knee, and hip joint position profiles go beyond 1.57 rad which is physiologically not relevant. The same model was simulated with LQR/LQE controller/observer [23] by linearizing the model at standing position and approximating delays with Pade. This modeling scheme reduced the ankle and knee joint profile deviation, but the hip joint profile is completely out of phase. Mughal and Iqbal in their recent study [24] proposed an LMI based control algorithm by combining the *H*_2_ and *H*_∞_ optimization. The results reported showed an improvement over the earlier studies, however, the joint profiles can still be seen with undesired deviation and oscillations. The authors concluded that a nonlinear feedback term with optimal linear control gains may lead to a more appropriate control law for physiologically relevant movement coordination. Keeping in view this statement our research aims to overcome the trajectory tracking problems by taking into account all nonlinearities of the biomechanical model and designing a nonlinear observer-based control for the biomechanical STS movement. Additionally, our simulation scheme compensates for delays without increasing the system’s order.

In our previous studies [25–27] we simulated the single-link biomechanical model for postural stability. We have shown how the nonlinear observer-based control helps analyze the postural stability in the presence of proprioceptive feedback delays and, input and measurement noises. In our current study, we aim at extending the rigorous mathematical paradigm to a four-segment biomechanical model with force, velocity, and position feedback involving physiological latencies. In particular, we aim to investigate the mechanism of STS movement in the human neuro-musculoskeletal system through in-depth analysis of a multi-segment biomechanical model using physiologically motivated nonlinear observer-based control. The multi-segment model presented in this paper represents the musculoskeletal-proprioceptive system. It includes the proprioceptive feedback latency of the motor servo system, sensors, and measurement noises. The model additionally considers the muscle impedance that includes the active torque and passive stiffness. The high-gain observer-based feedback linearization control scheme represents our neurophysiological controller, which mimics the behavior of CNS in controlling the STS movement. The major advantage of a high-gain observer (HGO) is its robustness against large perturbations and uncertainties. The design process for HGO is very simple; the observer gain is computed on the basis of a positive constant which must be chosen as small as possible in order to provide a quick state estimation. In the high-gain design, the observer bandwidth is tuned to obtain the desired stability/robustness properties. However, this comes at the cost of peaking which is overcome by saturating the control input during the transient state. The beauty of feedback linearization is that it cancels out the system’s nonlinearities by calculating a suitable control input that contains the nonlinear feedback gains, leaving behind the linear system with exact dynamics [28]. Unlike the Jacobean linearization, the transformed linear system is valid for all functional regions [29]. We augment feedback linearization with *H*_2_ to calculate the linear gains of the system. *H*_2_ minimizes the power gain of the system, the effects of disturbance, and noises. The physiologists [30,31] reported that the STS task depends upon the kinetic variables e.g. ground-reaction-forces (GRF), and kinematic variables such as center-of-mass (COM), center-of-pressure (COP), and head-position (HP). The physiological variables GRF and COM based controller architecture achieve physiologically correct and improved movement coordination for biomechanical STS tasks [32]. We optimize the state and input weighting matrices by penalizing them with physiological variables GRF and COM. Our emphasis here is to build a comprehensive mathematical framework for stability analysis and control synthesis that can be applied in the future to more in-depth models.

Our central hypothesis states that for detailed analysis and accurate simulations of sit-to-stand motion, the modeling scheme must consider the system’s nonlinearities. The biomechanical model contains the inertial, Coriolis, and gravitational components with sine and cosine terms. The linear controllers assume each joint to be independent and consider the inertia seen by each joint as a constant. This approximation results in non-uniform damping leading to undesirable deviations in joint position profiles. The linear approximation design approach is simple and sometimes it works, however, it might impair the original characteristics of the nonlinear system which may lead to inaccuracy or false conclusion. Thus, our statement on the subject research is that the nonlinear control scheme based on the high-gain observer and feedback linearization presents a much better emulation of CNS as compared to standard linear control strategies reported in the literature. Besides improving our understanding of the neurophysiological mechanism in the body, comprehensive models like the one described in this study are indispensable for examining pathological behavior resulting from unknown conditions.

## 2. Methods

In this section, the modeling and control-synthesis framework of the four-segments biomechanical model for STS task is described.

### 2.1. Multi-segments biomechanical model

**Fig 1:**
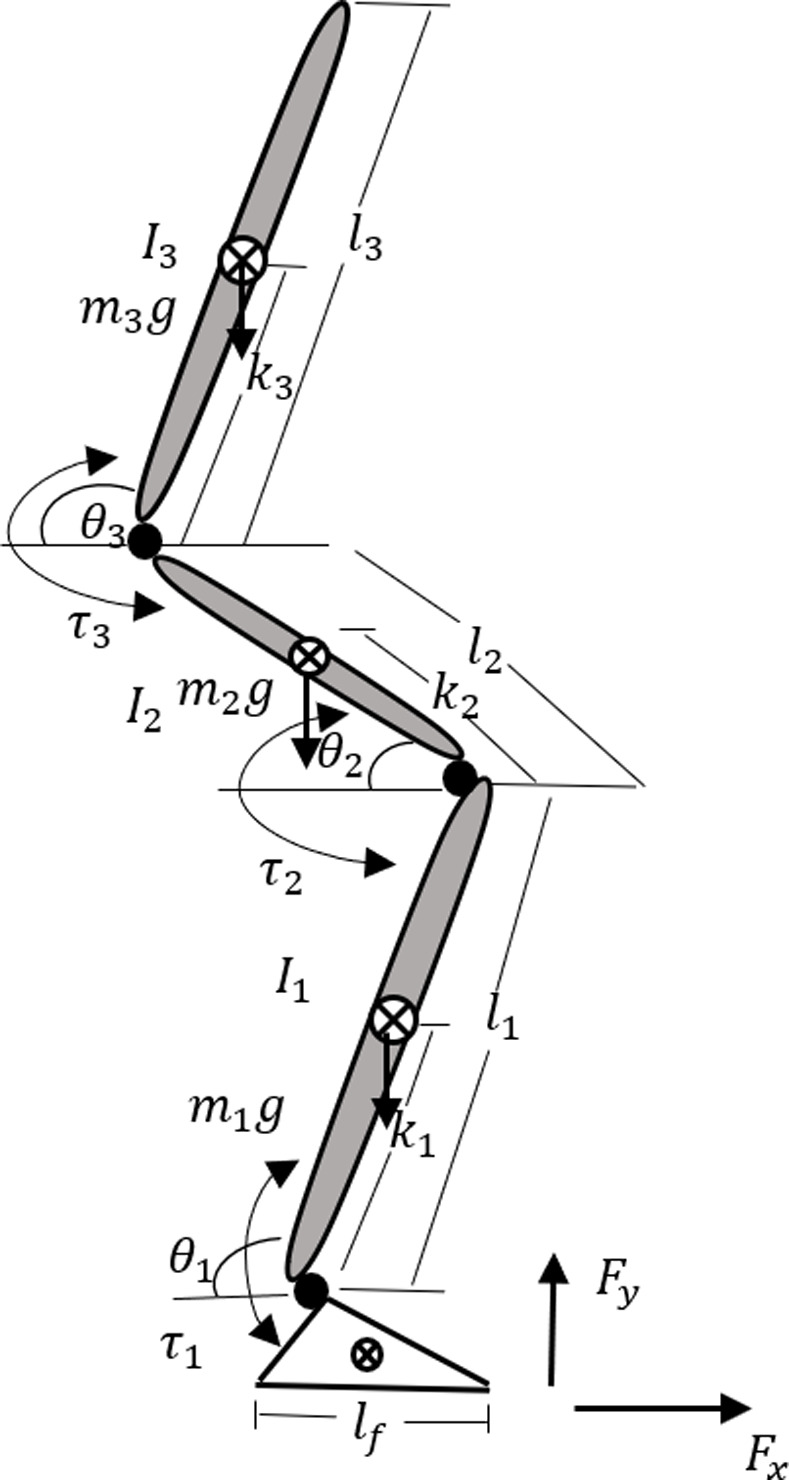
The layout of the 4-segments biomechanical model in the sagittal plane.

Keeping in view the STS task, the mechanics of the human body are modeled as 4-planer rigid body segments connected by a single degree-of-freedom (DOF) as shown in Fig 1. The multi-segment structure is assumed to contain skeletal, muscular, and sensory subsystems. The 4-segments represent the bilateral arrangement of feet, shank, thighs, and head-arm-trunk (HAT), approximating a total of 6-DOF in the sagittal plane mechanics. The foot length represents the base-of-support (BOS) in the anterior-posterior direction. The foot length is represented by *l*_*f*_ and its mass by *m*_*f*_. The shank, thighs, and HAT can rotate about the ankle, knee, and hip joints. *τ*_*i*_ (*i* = 1,2,3) represents the torque at these joints. The *F*_*x*_ and *F*_*y*_ are ground-reaction-forces acting on BOS in horizontal and vertical directions, respectively. The angular displacement variable *θ*_*i*_ represents the leg, thigh, and torso segments position with respect to the ground frame of reference. In Fig 1 *m*_*i*_, *l*_*i*_ and *l*_*i*_ represent the segments’ mass, length, and inertia, respectively. The parameter *k*_*i*_ represents the location of COM of each segment. To maintain the dynamic stability during STS, the BOS must encompass both COM and COP. The CNS commands from cerebral-cortex or reflex loop induce the muscle forces which then translate into ankle, knee, and hip joint torque actuation. The human postural and voluntary movements e.g. STS encompasses both active and passive mechanism [33]. The induced torques at all three joints are the sum of feedforward and feedback components. The feedforward torques represent the task-specific CNS commands. The feedback torques represent the CNS response to proprioceptive feedback and are produced by intrinsic stiffness or viscosity of muscle-tendon complexes, thus mimic the lower level motor-servo feature.

### 2.2. Open-loop model dynamics

The multi-segments biomechanical model shown in Fig 1 exhibits nonlinear dynamics and is illustrated by the following matrix differential equation.

$$D(\theta )\vec{\ddot{\theta }}+H(\theta ,\dot{\theta })\vec{\dot{\theta }}+G(\theta )=\vec{\tau }$$ (1)

where *D* (*θ*) ∈ *R*^3×3^, $H(\theta ,\dot{\theta })\in R^{3\times 3}$ and *G* (*θ*) ∈ *R*^3×1^, represent the inertial, Coriolis and gravitational components of torque respectively defined by Iqbal and Pai [34] given as:

$$D(\theta )=\left[\begin{array}{ccc} d_{11} & d_{12}\cos(\theta_{1}-\theta_{2}) & d_{13}\cos(\theta_{1}-\theta_{3})\\ d_{12}\cos(\theta_{1}-\theta_{2}) & d_{22} & d_{23}\cos(\theta_{2}-\theta_{3})\\ d_{13}\cos(\theta_{1}-\theta_{3}) & d_{23}\cos(\theta_{2}-\theta_{3}) & d_{33} \end{array}\right]$$

$$H(\theta ,\dot{\theta })=\left[\begin{array}{ccc} 0 & d_{12}\theta_{5}\sin(\theta_{1}-\theta_{2}) & d_{13}\theta_{6}\sin(\theta_{1}-\theta_{3})\\ -d_{12}\theta_{4}\sin(\theta_{1}-\theta_{2}) & 0 & d_{23}\theta_{6}\sin(\theta_{2}-\theta_{3})\\ -d_{13}\theta_{4}\sin(\theta_{1}-\theta_{3}) & -d_{23}\theta_{5}\sin(\theta_{2}-\theta_{3}) & 0 \end{array}\right]$$

$$G=g{\left[f_{1}\cos(\theta_{1})\;f_{2}\cos(\theta_{2})\;f_{3}\cos(\theta_{3})\right]}^{T}$$

where $d_{11}=m_{1}k_{1}^{2}+(m_{2}+m_{3})l_{1}^{2}+I_{1},d_{22}=m_{2}k_{2}^{2}+m_{3}l_{2}^{2}+I_{2},d_{33}=m_{3}k_{3}^{2}+I_{3},d_{12}=f_{2}l_{1},d_{13}=f_{3}l_{1}{,}^{\infty }d_{23}=f_{3}l_{2},f_{1}=m_{1}k_{1}+(m_{2}+m_{3})l_{1},f_{2}=m_{2}k_{2}+m_{3}l_{2}+m_{3}l_{2},f_{3}=m_{3}k_{3}$

The biomechanical parametric values are based on average anatomical proportions and are taken from the study by De Leva [35] given in Table 1. Zatsiorsky et al. [36] calculated the relative body segments’ masses, COM positions, and radii of gyration of college-aged Caucasian males and females using the gamma-ray scanning technique. This data was rarely used for the analysis of biomechanical movements since the bony landmarks were used as reference points for calculating segment lengths and locating COM. De Leva [35] adjusted the mean relative COM positions and radii of gyration based on carefully selected sources of anthropometric data. They referenced the adjusted data to the joint centers and/or other widely used landmarks, rather than the original bony landmarks. We used the biomechanical parametric values defined by De Leva [35] and used by many researchers [5,21,24].

**Table 1:** Biomechanical parametric values used in simulations.

Physical Parameter	Notation	Value
Gravitational moment components	*f*_1_, *f*_2_, *f*_3_	[26.6,22.52 13.84]*kgm*
Segment masses	*m*_1_,*m*_2_,*m*_3_	[6,14,13.20,44.75]*kg*
Segment moment of inertia	*I*_1_, *I*_2_, *I*_3_	[0.105,0.256,7.53]*kgm*^2^
Segment length	*l*_1_, *l*_2_, *l*_3_	[0.433,0.431,0827]*m*
Segment distance from center of mass (COM)	*k*_1_, *k*_2_, *k*_3_	[0.246,0.246,0.309]*m*
Foot mass and length	*m*_*f*_, *l*_*f*_	1.91*kg*,0.27*m*
Ankle heel, height, ankle-foot COM	*a*, *b*, *c*	[0.05,0.07,0.08]*m*
Gravitational acceleration	*g*	9.8*m*/*s*^2^

In Eq 1 the torque $\vec{\tau }={\left[\tau_{1}-\tau_{2}\tau_{2}-\tau_{3}\tau_{3}\right]}^{T}$ reflects the input torque at the ankle, knee, and hip joints. Furthermore, the torque at each joint is the sum of *τ*_*ff*_ (feed-forward) and *τ*_*fb*_ (feedback) components. The *τ*_*ff*_ represents the task-specific commands from CNS and ensures to retain static balance in the upright position as well as at discrete points along the reference trajectory *ϑ*_*ref*._ The *τ*_*fb*_ reflects the passive viscoelasticity in the muscle-tendons surrounding the joint. CNS produces this torque as a result of muscular stretches necessary for the movement. We define the angular displacement $\vec{\theta }$ and angular velocity $\vec{\dot{\theta }}$ as our state variables. The nonlinear dynamic equation defined in Eq 1 can be re-written in the following inverse kinematics form.

$$\vec{\ddot{\theta }}=D{\left(\theta \right)}^{-1}[\vec{\tau }-H(\theta ,\dot{\theta })\vec{\dot{\theta }}-G(\theta )]$$ (2)

The state variables of the model are defined as:

$$x={\left[\theta_{1}\;\theta_{2}\;\theta_{3}\;\theta_{4}\;\theta_{5}\;\theta_{6}\right]}^{T}$$ (3)

where *θ*_1_, *θ*_2_, *θ*_3_ are position state variables and *θ*_4_, *θ*_5_, *θ*_6_ are velocity state variables.

The model is represented in nonlinear state-space form as:

$${\dot{x}}_{1}=x_{4},{\dot{x}}_{2}=x_{5},{\dot{x}}_{3}=x_{6}$$ (4)

$$\left[\begin{array}{c} {\ddot{\theta }}_{1}\\ {\ddot{\theta }}_{2}\\ {\ddot{\theta }}_{3} \end{array}\right]=\left[\begin{array}{c} {\dot{x}}_{4}\\ {\dot{x}}_{5}\\ {\dot{x}}_{6} \end{array}\right]=\left[D^{-1}\right]\left[-H\left[\begin{array}{c} x_{4}\\ x_{5}\\ x_{6} \end{array}\right]-G+\vec{\tau }\right]$$

Eq 4 depicts the biomechanical model in terms of position state variables *x*_1_,*x*_2_,*x*_3_, and velocity state variables ${\dot{x}}_{4}$, ${\dot{x}}_{5}$, ${\dot{x}}_{6}$. For simplicity and generalization of equations, we will use the following notations for calculations. State and input equations are function of *x*, thus we can rewrite Eq 4 in the following form.

$$\vec{\dot{x}}=f(x)+g(x)\vec{u}$$ (5)

$$\vec{y}=h(x)$$

where *f*(*x*) represents the nonlinear state equations and *g*(*x*) is the coefficient of input torque $\vec{u}$ at the ankle, knee, and hip joints, respectively. *h*(*x*) represents the output equation. These terms can be further elaborated as:

$$f=\left[\begin{array}{c} x_{4}\\ x_{5}\\ x_{6}\\ \alpha_{1}(x_{1},x_{2},x_{3},x_{4},x_{5},x_{6})\\ \alpha_{2}(x_{1},x_{2},x_{3},x_{4},x_{5},x_{6})\\ \alpha_{3}(x_{1},x_{2},x_{3},x_{4},x_{5},x_{6}) \end{array}\right],\;g=\left[\begin{array}{ccc} 0 & 0 & 0\\ 0 & 0 & 0\\ 0 & 0 & 0\\ \beta_{41} & \beta_{42} & \beta_{43}\\ \beta_{51} & \beta_{52} & \beta_{53}\\ \beta_{61} & \beta_{62} & \beta_{63} \end{array}\right]$$ (6)

The position of the segments i.e., ankle, knee, and hip angles are of interest for STS movement, so we define our output equation as:

$$\vec{y}={\left[x_{1}\;x_{2}\;x_{3}\right]}^{T}$$ (7)

### 2.3. Physiological feedback latencies

The joint angles suffer physiological latencies in transmissions to CNS. Sinkjaer and his colleagues [37] conducted an experimental study on humans and reported 50 ms ankle flexor/extensor pathway delay. Iqbal and Anindo Roy [5] further elaborated on these physiological delays and defined the delays at each joint. According to Jiang et al. [38] the delay between the motor operation and peak force production can vary from 20ms-40ms. Here we assume that the delay at each joint represents the proprioceptive feedback latency. These feedback delays are the same as the delays due to MS and GTO. We define the delays at the ankle, knee, and hip joint position and velocity in Table 2 as defined by Iqbal and Anindo Roy [5].

**Table 2:** Feedback delays at ankle, knee, and hip joints.

Joint	Physical Quantity	Delay (ms)
Ankle	position, velocity	30
Knee	position, velocity	15
Hip	position, velocity	10

Table 2 shows that the hip joint delay is the least. The ankle joint suffers the longest delay due to an increase in the transmission path to CNS. Now we can represent our delayed output states as:

$$\vec{y}=\left[\begin{array}{c} x_{1}(t-t_{1})\\ x_{2}(t-t_{2})\\ x_{3}(t-t_{3}) \end{array}\right]$$ (8)

where *t*_1_ = 30ms, *t*_2_ = 15ms, and *t*_3_ = 10ms. We define $\left(t-t_{1})≅\varsigma_{1}(t\right)$ for simplicity. We will use the same notation for other delayed states as well. The physiological feedback delays cannot be controlled, we will estimate and compensate them with the high-gain observer.

## 3. Controller design

### 3.1. Feedback linearization tracking control

The biomechanical model defined in Eq 5 is nonlinear and open-loop unstable system. We need to design a control law that cancels the nonlinearities in the system, as well as the outputs track the reference trajectories to maintain stable body movement during STS transfer. Feedback linearization is a well-known technique for nonlinear systems design and analysis. The core concept of this method is to transform the nonlinear dynamics of a system algebraically into fully or partially linearized ones so that it can be combined with linear control techniques. The feedback linearization control-law comprises two parts. One part cancels the system’s nonlinearities, and the second part controls the subsequent linear system. Unlike Jacobian linearization, the control law is valid for all operating points. In the current study, we use feedback linearization in combination with *H*_2_ control technique. The *H*_2_ calculates linear gains of the system. The model described in Eq 5 must appease the assumptions defined by Hassan Khalil [29] and Zak [28]. The functions *f*(*x*), *g*(*x*), *h*(*x*) must be smooth and differentiable continuously in the domain *D* ∈ *R*^*n*^. The function *f*(0) = 0, and the mapping *f*:*D → R*^*n*^ and *g*:*D → R*^*n*^ are vector fields, that contain the origin *x* = 0, *u* = 0. To define the relative degree of output, we differentiate the output continually until the input *u* appears in the output. The relative degree comes out to be [*r*_1_ *r*_2_ *r*_3_] = [2 2 2]. It is desired that the output tracks the reference signals asymptotically.

#### 3.1.1. Reference trajectories

Mughal and Iqbal [39] conducted an experimental study on STS movement and generated ankle, knee, and hip joint position profiles during STS transfer. To simulate the point-to-point movement, we use a reference model for trajectory generation whose output resembles all aspects of the experimental study by Mughal and Iqbal [39]. The general form of individual reference trajectory for STS transfer is given as:

$$\vartheta_{ref}(t)=x_{i}+(x_{f}-x_{i}).x_{R}(t)$$ (9)

where *x*_*i*_ and *x*_*f*_ represent the initial and final position of segment posture. *x*_*R*_(*t*) can be calculated from the output of a third-order open-loop stable linear system. The trajectory model is given as:

$${\vec{\dot{x}}}_{R}(t)=A_{R}{\vec{x}}_{R}(t)$$ (10)

where $A_{R}=\left[\begin{array}{ccc} 0 & 1 & 0\\ 0 & 0 & 1\\ 0 & -\mu & -\rho \end{array}\right]$. *x*_*R*_(*t*) can also be obtained from a sigmoid function $x_{R}(t)=\frac{1-e^{-ct}}{1+e^{-ct}}$. As t → 0 the *x*_*R*_(*t*) → 1. The initial conditions for ankle, knee, and hip are $\left[\begin{array}{c} \frac{\pi }{2}\\ 0\\ \frac{\pi }{2} \end{array}\right]$. The reference trajectories *ϑ*_*ra*_(*t*), *ϑ*_*rk*_(*t*) and *ϑ*_*rh*_(*t*) are generated by employing these initial conditions.

#### 3.1.2. Tracking control

In order for the output to track the reference trajectories asymptotically, the reference angles must fulfill the assumptions employed by Hassan Khalil [29,40].

- *ϑ*_*ra*_(*t*), *ϑ*_*rk*_(*t*), *ϑ*_*rh*_(*t*), and its derivatives up to the relative degree of system *r*^*n*^ are bounded for *t* ≥ 0. The *n*^*th*^ derivative of *ϑ*_*ra*_(*t*), *ϑ*_*rk*_(*t*) and *ϑ*_*rh*_(*t*) is a piecewise continuous function of *t*.
- The signals *ϑ*_*ra*_(*t*), *ϑ*_*rk*_(*t*), *ϑ*_*rh*_(*t*),------ $\vartheta_{ra}^{n}(t),\;\vartheta_{rk}^{n}(t),\text{and}\vartheta_{rh}^{n}(t)$ are obtainable on-line.

Since we use the state model defined in Eq 10 for reference generation, so all the reference angles and their derivatives fulfill the required assumptions. With the system’s relative degree 2, we desire the output *y* to track the reference angles with bounded derivatives ${\dot{\vartheta }}_{ref}(t)$ and ${\ddot{\vartheta }}_{ref}(t)$. We introduce delays in states and references. The system has the following dynamic equations.


$$\left[\begin{array}{c} e_{1}\\ e_{2}\\ e_{3}\\ e_{4}\\ e_{5}\\ e_{6} \end{array}\right]=\left[\begin{array}{c} x_{1}(\varsigma_{1}(t))-\vartheta_{ra}(\varsigma_{1}(t))\\ x_{2}(\varsigma_{2}(t))-\vartheta_{rk}(\varsigma_{2}(t))\\ x_{3}(\varsigma_{3}(t))-\vartheta_{rh}(\varsigma_{3}(t))\\ x_{4}(\varsigma_{1}(t))-{\dot{\vartheta }}_{ra}(\varsigma_{1}(t))\\ x_{5}(\varsigma_{2}(t))-{\dot{\vartheta }}_{rk}(\varsigma_{2}(t))\\ x_{6}(\varsigma_{3}(t))-{\dot{\vartheta }}_{rh}(\varsigma_{3}(t)) \end{array}\right]$$ (11)

The design goal is to ensure that the vector *e* = col(*e*_1_,*e*_2_,…,*e*_6_) = (*x*(*ς*)– *ϑ*_*ref*_(*ς*)) is bounded for all *t* ≥ 0 and *e* → 0 as *t* → ∞. The boundness of *e* implies the boundness of states since *ϑ*_*ref*_ is already bounded.


$$\begin{array}{l} {\dot{e}}_{1}=e_{4}\\ {\dot{e}}_{2}=e_{5}\\ {\dot{e}}_{3}=e_{6}\\ {\dot{e}}_{4}=\alpha_{1}(x(\varsigma (t)))+\beta_{41}(x(\varsigma (t)))u_{1}(t)+\beta_{42}(x(\varsigma (t)))u_{2}(t)+\beta_{43}(x(\varsigma (t)))u_{3}(t)-{\ddot{\vartheta }}_{ra}(\varsigma_{1}(t))\\ {\dot{e}}_{5}=\alpha_{2}(x(\varsigma (t)))+\beta_{51}(x(\varsigma (t)))u_{1}(t)+\beta_{52}(x(\varsigma (t)))u_{2}(t)+\beta_{53}(x(\varsigma (t)))u_{3}(t)-{\ddot{\vartheta }}_{rk}(\varsigma_{2}(t))\\ {\dot{e}}_{6}=\alpha_{3}(x(\varsigma (t)))+\beta_{61}(x(\varsigma (t)))u_{1}(t)+\beta_{62}(x(\varsigma (t)))u_{2}(t)+\beta_{63}(x(\varsigma (t)))u_{3}(t)-{\ddot{\vartheta }}_{rh}(\varsigma_{3}(t)) \end{array}$$ (12)

The system is fully invertible. For the convenience of notation, we redefine the terms in Eq 12 as:

$$\begin{array}{l} \alpha =\left[\begin{array}{c} \alpha_{1}\;(x(\varsigma (t)))\\ \alpha_{2}\;(x(\varsigma (t)))\\ \alpha_{3}\;(x(\varsigma (t))) \end{array}\right],\;{\ddot{\vartheta }}_{ref}=\left[\begin{array}{c} {\ddot{\vartheta }}_{ra}\;(\varsigma_{1}\;(t))\\ {\ddot{\vartheta }}_{rk}\;(\varsigma_{2}\;(t))\\ {\ddot{\vartheta }}_{rh}\;(\varsigma_{2}\;(t)) \end{array}\right],\\ \beta \left[\begin{array}{ccc} \beta_{41}\;(x(\varsigma (t))) & \beta_{42}\;(x(\varsigma (t))) & \beta_{43}\;(x(\varsigma (t)))\\ \beta_{51}\;(x(\varsigma (t))) & \beta_{52}\;(x(\varsigma (t))) & \beta_{53}\;(x(\varsigma (t)))\\ \beta_{61}\;(x(\varsigma (t))) & \beta_{62}\;(x(\varsigma (t))) & \beta_{63}\;(x(\varsigma (t))) \end{array}\right] \end{array}$$ (13)

From Eq 12 we define the following matrices for ease of notation only.


$$A=\left[\begin{array}{cccccc} 0 & 0 & 0 & 1 & 0 & 0\\ 0 & 0 & 0 & 0 & 1 & 0\\ 0 & 0 & 0 & 0 & 0 & 1\\ 0 & 0 & 0 & 0 & 0 & 0\\ 0 & 0 & 0 & 0 & 0 & 0\\ 0 & 0 & 0 & 0 & 0 & 0 \end{array}\right],B=\left[\begin{array}{ccc} 0 & 0 & 0\\ 0 & 0 & 0\\ 0 & 0 & 0\\ 1 & 1 & 1\\ 1 & 1 & 1\\ 1 & 1 & 1 \end{array}\right]\;\text{and}\;u=\left[\begin{array}{c} u_{1}(t)\\ u_{2}(t)\\ u_{3}(t) \end{array}\right]$$

Using *A*,*B*,*u*, and notation defined in Eq 13 we can represent Eq 12 in the following form.


$$\vec{\dot{e}}=A\vec{e}+B[\alpha (\vec{x}(\varsigma (t)))+\beta (\vec{x}(\varsigma (t)))\vec{u}-{\vec{\ddot{\vartheta }}}_{ref}(\varsigma (t))]$$ (14)

Now we define the state feedback law as:

$$\vec{u}={\left[\beta (\vec{x}(\varsigma (t)))\right]}^{-1}[-\alpha (\vec{x}(\varsigma (t)))+{\vec{\ddot{\vartheta }}}_{ref}(\varsigma (t))+v]$$ (15)

where $v=-K\vec{e}\in R^{3\times 6}$ and *K* define the linear feedback gains. Inserting Eqs 15 in 14 linearizes the system by canceling all the nonlinearities. The system’s closed-loop equation now takes the form:

$$\vec{\dot{e}}=(A-BK)\vec{e}$$ (16)

We calculate the linear feedback gains defined by $v=-K\vec{e}$ in Eq 15 using *H*_2_ control technique. *H*_2_ control is an optimal and robust control method that bounds the system’s power gain and significantly reduces the impact of noise disturbance, discussed in detail by Doyle [41]. The linear model of the plant defined in Eq 5 with input and measurement noise is given as:

$$\vec{\dot{x}}(t)=\overset{⌣}{A}x(t)+B_{u}u(t)+B_{w}w(t)$$ (17)

The measured and controlled outputs are given as:

$$\left[\begin{array}{c} \vec{m}(t)\\ \vec{y}(t) \end{array}\right]=\left[\begin{array}{c} c_{m}\\ c_{y} \end{array}\right]x(t)+\left[\begin{array}{cc} D_{mu} & D_{mw}\\ D_{yu} & D_{yw} \end{array}\right]\cdot \left[\begin{array}{c} u(t)\\ w(t) \end{array}\right]$$ (18)

*m*(*t*) represents the measurement and *y*(*t*) is the output to be minimized. $w(t)=\left[\begin{array}{c} n(t)\\ z(t) \end{array}\right]$ represents the white noise disturbance. *n*(*t*) is the input process noise and *z*(*t*) is the output measurement noise and are represented by the following covariance matrices.


$$E(n(t)n^{T}(t+\tau ))=S_{n}\delta (\tau )$$ (19)

$$E(z(t)z^{T}(t+\tau ))=S_{z}\delta (\tau )$$ (20)

The input process and output measurement noises defined in Eqs 19 and 20 are added at the plant input and output respectively and will accommodate for noise present at the plant input or output. The input and output noises have a signal power *S*_*n*_*δ*(*τ*) = 1*μW* and *S*_*z*_*δ*(*τ*) = 4*nW* respectively. The system defined in Eqs 17 and 18 is combined into *p* matrix given as:

$$p=\left[\begin{array}{ccc} \overset{⌣}{A} & B_{w} & B_{u}\\ C_{y} & D_{yw} & D_{yu}\\ C_{m} & D_{mw} & D_{mu} \end{array}\right]$$ (21)

*D*_*yw*_ and *D*_*mu*_ are zero matrices while *D*_*yu*_ and *D*_*mw*_ must be full rank. *C*_*y*_ and *D*_*yu*_ represent the states and input weighting functions and are scaled for states and input optimization. These matrices are usually scaled arbitrarily using trial and error. However, we use the physiological variables in optimizing the cost functions. Mughal and Iqbal [32] reported the role of two physiological variables COM and GRF for optimal weights selection. In our current study, we use the same COM and GRF based optimization for calculating the state and input weighting matrices *C*_*y*_ and *D*_*yu*_ (given in the appendix). These physiological variables impose constraints on STS movement coordination and integration.

The whole system defined in Eq 21 must follow the assumptions employed by Doyle [41,42]. A 6^th^ order compensator equation is obtained after solving the following two Riccati equations.


$$S\overset{⌣}{A}+{\overset{⌣}{A}}^{T}S-S(B_{u}B_{u}^{T})S+C_{y}^{T}C_{y}=0$$ (22)

$$Q\overset{⌣}{A}+{\overset{⌣}{A}}^{T}Q-Q(C_{m}^{T}C_{m})Q+B_{w}B_{w}^{T}=0$$ (23)

The compensator equation is given as:

$$\begin{array}{l} {\vec{\dot{x}}}_{c}={\overset{⌣}{A}}_{c}x_{c}(t)+B_{c}m(t)\\ \;\cdots \vec{u}(t)=C_{c}x_{c} \end{array}$$ (24)

${\overset{⌣}{A}}_{c}$ is a 6^th^ order matrix with 3 position and 3 velocity states, *B*_*c*_ ∈ *R*^6×3^ and *C*_*c*_ ∈ *R*^3×6^ are observer and controller gain matrices, respectively. These gains constitute the linear part of HGO and feedback linearization controller. We represent *C*_*c*_ = *K* and *B*_*c*_ = *L* in our simulation for STS movement.

The closed-loop system defined in Eq 16 contains the delayed states. The control objective is that the states track the reference trajectories with minimum error. We need the control law to minimize the tracking error, compensate for delays, and diminish the model uncertainties. To accomplish this, the control law with output feedback is required. The state estimates can be obtained from high-gain observer explain in section 3.2.

### 3.2. High-gain observer design

The high-gain observer plays a vital role in the feedback control design of nonlinear systems owing to its two main features. It decays the estimation error to a very small value *0*(*ε*) with a time interval reducing to zero as *ε* decreases. The second main feature is its robustness against uncertain nonlinear functions. Hassan Khalil [43] reported the performance recovery feature of the high-gain observer against time-varying delays. Motivated by all these features we design a high-gain observer for state estimation of our model. The control signal defined in Eq 15 is a locally Lipschitz state feedback controller. To execute this controller with output feedback the high-gain observer is given as:

$$\begin{array}{l} {\dot{\hat{e}}}_{1}={\hat{e}}_{4}+\frac{L_{1}}{\varepsilon }(e_{1}-{\hat{e}}_{1})\\ {\dot{\hat{e}}}_{2}={\hat{e}}_{5}+\frac{L_{2}}{\varepsilon }(e_{2}-{\hat{e}}_{2})\\ {\dot{\hat{e}}}_{3}={\hat{e}}_{6}+\frac{L_{3}}{\varepsilon }(e_{3}-{\hat{e}}_{3})\\ {\dot{\hat{e}}}_{4}=\alpha_{1}(\hat{e},\vartheta_{ref})+\beta_{41}(\hat{e},\vartheta_{ref})u_{1}(t)+\beta_{42}(\hat{e},\vartheta_{ref})u_{2}(t)+\beta_{43}(\hat{e},\vartheta_{ref})u_{3}(t)-{\ddot{\vartheta }}_{ra}+\frac{L_{4}}{\varepsilon^{2}}(e_{1}-{\hat{e}}_{1})\\ {\dot{\hat{e}}}_{5}=\alpha_{2}(\hat{e},\vartheta_{ref})+\beta_{51}(\hat{e},\vartheta_{ref})u_{1}(t)+\beta_{52}(\hat{e},\vartheta_{ref})u_{2}(t)+\beta_{53}(\hat{e},\vartheta_{ref})u_{3}(t)-{\ddot{\vartheta }}_{rk}+\frac{L_{5}}{\varepsilon^{2}}(e_{2}-{\hat{e}}_{2})\\ {\dot{\hat{e}}}_{6}=\alpha_{3}(\hat{e},\vartheta_{ref})+\beta_{61}(\hat{e},\vartheta_{ref})u_{1}(t)+\beta_{62}(\hat{e},\vartheta_{ref})u_{2}(t)+\beta_{63}(\hat{e},\vartheta_{ref})u_{3}(t)-{\ddot{\vartheta }}_{rh}+\frac{L_{5}}{\varepsilon^{2}}(e_{3}-{\hat{e}}_{3}) \end{array}$$ (25)

The correction terms in HGO are $\left(e_{1}-{\hat{e}}_{1}),(e_{2}-{\hat{e}}_{2})\;\text{and}\;(e_{3}-{\hat{e}}_{3}\right)$. Eq 25 shows that ${\hat{e}}_{1},{\hat{e}}_{2},{\hat{e}}_{3}$ estimate *e*_1_,*e*_2_,*e*_3_ and compensate for the delays in states. The observer equation can be written in the following form, for convenience of notation:

$$\vec{\dot{\hat{e}}}=A\vec{\hat{e}}+B[\alpha (\hat{e},\vartheta_{ref})+\beta (\hat{e},\vartheta_{ref})\vec{u}-{\vec{\ddot{\vartheta }}}_{ref}]+H(\varepsilon )(\vec{e}-\vec{\hat{e}})$$ (26)

where pair (*A*, *B*) represents the chain of integrators as defined earlier. $\hat{x}(t)=\hat{e}+\vartheta_{ref}\in R_{x}$ and $H(\varepsilon )={\left[\frac{L_{1}}{\varepsilon }\;\frac{L_{2}}{\varepsilon }\;\frac{L_{3}}{\varepsilon }\;\frac{L_{4}}{\varepsilon }\;\frac{L_{5}}{\varepsilon }\;\frac{L_{6}}{\varepsilon }\right]}^{T}\in R^{6\times 6}$ where *ε* is a very small value, we use *ε* = 0.04, *L*_*i*_(*i* = 1…6) are the linear feedback gains calculated from *H*_2_ control technique defined by Eq 24 as *B*_*c*_ = *L*. The difference in initial conditions of controller and observer i.e., $x_{1}(0)=\frac{\pi }{2},{\hat{x}}_{1}(0)=0,x_{3}(0)=\frac{\pi }{2},{\hat{x}}_{3}(0)=0$ induces peaking of the order of $O\left(\frac{1}{\varepsilon }\right)$ causing the closed-loop system to destabilize. Saturating the control signal outside the compact set of interest creates a buffer that prevents the plant from peaking [40].

The linear feedback gain *K* is calculated using *H*_2_ control technique such that (*A*—*BK*) in Eq 16 is Hurwitz. Thus *e*(*t*) is bounded where $\underset{t\to \infty }{\text{lim}}e(t)=0$. Consequently, *x*(*ς*(*t*)) = *e*+*ϑ*_*ref*_ is bounded. The positive definite solution *P*_1_ of the Lyapunov equation *P*_1_(*A*—*BK*) + (*A*—*BK*)^*T*^*P*_1_ = -*Q*_1_, where *Q*_1_ = *I*, can generate the compact set of closed-loop system [40]. Let Ψ = (*e*^*T*^*P*_1_*e* ≤ *S*) is a positive invariant compact set of Eq 16 for any *S* > 0. We choose *S* > 0 such that for every *e* ∈ Ψ, *x*(*ς*(*t*)) = *e* + *ϑ*_*ref*_ ∈ *R*_*x*_. Since all initial conditions belong to Ψ = (*e*^*T*^*P*_1_*e* ≤ 6). We take $M_{i}>max_{e\in \Psi }\left|e_{i}\right|$ for (*i* = 1….6), and saturate *α*(*x*), *β*(*x*),and *Ke* as follows:

$$\begin{array}{l} \;\alpha_{s}(e,\vartheta_{ref})=\alpha (e+\vartheta_{ref})|{}_{e_{i\to M_{i}sat(\frac{e_{i}}{M_{i}})}}\text{and}\\ \beta_{s}(e,\vartheta_{ref})=max\left\{\beta_{0},\;\beta (e+\vartheta_{ref})|{}_{e_{i\to M_{i}sat(\frac{e_{i}}{M_{i}})}}\right\}\\ \;Ke=\sum_{i=1}^{6}K_{i}M_{i}\text{sat}(\frac{e_{i}}{M_{i}}) \end{array}$$ (27)

After rigorous mathematical calculations we get $max_{e\in \Psi }\left|e_{1}\right|=8.35,max_{e\in \Psi }\left|e_{2}\right|=8.538,max_{e\in \Psi }\left|e_{3}\right|=9.74,max_{e\in \Psi }\left|e_{4}\right|=1.435,max_{e\in \Psi }\left|e_{5}\right|=1.404,max_{e\in \Psi }\left|e_{6}\right|=1.2317.$ We saturate *e*_1_ at ±12, *e*_2_at ±13, *e*_3_ at ±15, e_4_ at ±6, *e*_5_ at ±5, *e*_6_ at ±3. The functions *α*_*s*_, *β*_*s*_ are bounded globally in *e*, *α*_*s*_ = *α*, and *β*_*s*_ = *β* for *e* ∈ Ψ and *β*_*s*_(e,*ϑ*_*ref*_) ≥ *β*_0_. $\sum_{i=1}^{6}K_{i}M_{i}\text{sat}(\frac{e_{i}}{M_{i}})=Ke$ for *e* ∈ Ψ. Thus the high-gain observer and output feedback controller are defined by modifying Eqs 15 and 26 as follows:

$$\vec{\dot{\hat{e}}}=A\vec{\hat{e}}+B[\alpha_{S}(\hat{e},\vartheta_{ref})+\beta_{S}(\hat{e},\vartheta_{ref})\vec{u}-{\vec{\ddot{\vartheta }}}_{ref}]+H(\varepsilon )(\vec{e}-\vec{\hat{e}})$$ (28)

$$\vec{u}=\beta_{s}^{-1}(\hat{e},\vartheta_{ref})\left[-\alpha_{s}(\hat{e},\vartheta_{ref})+\vec{\ddot{\vartheta }}-\sum_{i=1}^{6}K_{i}M_{i}\text{sat}(\frac{{\hat{e}}_{i}}{M_{i}})\right]$$ (29)

Inserting Eqs 29 in 28 simplifies the observer dynamics as given below.


$$\vec{\hat{\dot{e}}}=A\vec{\hat{e}}-B\sum_{i=1}^{6}K_{i}M_{i}\text{sat}(\frac{{\hat{e}}_{i}}{M_{i}})+H(\varepsilon )(\vec{e}-\vec{\hat{e}})$$ (30)

### 3.3. Feedforward control

The feedforward torques provide active control signals. The reference trajectories generate the feedforward torques. The nonlinear model defined in Eq 1 evaluated at the equilibrium standing position is equal to *τ*_*eq*_. Thus, these torques are due to gravitational components *G*(*θ*) given as:

$${\vec{\tau }}_{eq}={\left|g\cdot \left[\begin{array}{c} f_{1}\;cos\;(x_{1})+f_{2}\;cos\;(x_{2})+f_{3}\;cos\;(x_{3})\\ f_{2}\;cos\;(x_{2})+f_{3}\;cos\;(x_{3})\\ f_{3}\;cos\;(x_{3}) \end{array}\right]\right|}_{\vec{x}={\vec{x}}_{eq}}$$ (31)

The equilibrium torque ${\vec{\tau }}_{eq}$ is zero at the standing position and nonzero at any other position. The feedforward torque ${\vec{\tau }}_{ff}$ is calculated from reference trajectories employing the same gravitational component given as:

$${\vec{\tau }}_{ff}=g\cdot \left[\begin{array}{c} f_{1}\;cos\;(\vartheta_{ra})+f_{2}\;cos\;(\vartheta_{rk})+f_{3}\;cos\;(\vartheta_{rh})\\ f_{2}\;cos\;(\vartheta_{rk})+f_{3}\;cos\;(\vartheta_{rh})\\ f_{3}\;cos\;(\vartheta_{rh}) \end{array}\right]$$ (32)

The feedforward control represents muscle commands without feedback from joints (reflex action), so delay is not needed to be modeled here as it is a one-way command. The total torque to the nonlinear biomechanical model is the sum of feedforward and feedback torque defined in Eqs 29 and 32, respectively.

## 4. Simulation results

To determine the efficacy of our physiological nonlinear compensator for the biomechanical multi-segment model, we perform computer simulations of representative sit-to-stand movement. The nonlinear dynamic model in Eq 1 and the nonlinear observer and controller in Eqs 28 and 29 act as a closed-loop system with physiological feedback delays, sensors, and measurement noise in the loop. The initial position is sitting, and the intended position is an equilibrium standing position.

**Fig 2:**
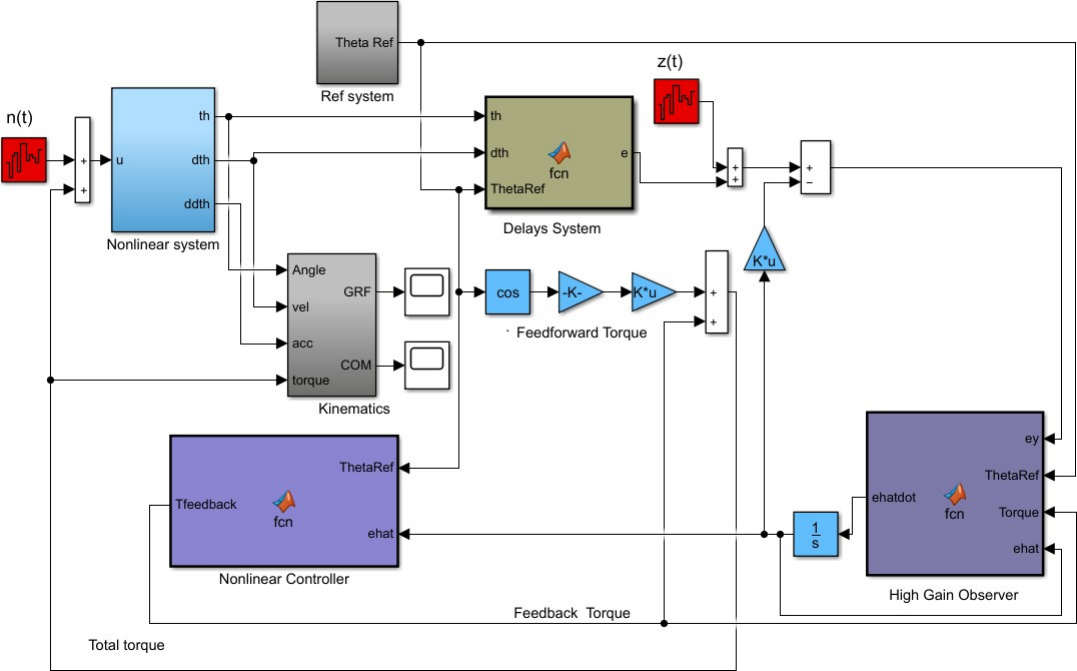
Sit-to-stand movement simulations developed in Simulink.

The overall simulation scheme built-in MATLAB/Simulink is represented by the block diagram shown in Fig 2. The block diagram comprises the nonlinear system block in which we implemented the nonlinear model represented by Eq 1. The reference system contains the reference trajectories based on experimental data [39] illustrated in Eqs 9 and 10. We further obtain the derivatives of reference trajectories from Eq 10. The reference trajectories generate the feedforward torque component which combines with the feedback torque component to compute the net joint torque. The delay system delays the states and references by different values given in Table 2. The kinematics block takes the position, velocity, acceleration, and torque as input and computes the kinematic variables COM, head position, and kinetic variable GRF. *n*(*t*) and *z*(*t*) represent the process and measurement white noise inputs to the system. The HGO is sensitive to measurement noise since it estimates the output derivatives. When the noise corrupts the output, it can have a serious effect on the state estimates. However, this problem does not arise if the noise frequency is low [40]. The measurement white noise has a signal power of 4*nW* with sample time 0.01s and thus does not affect the state estimates. The controller block implements Eq 29 and generates the control signal (feedback torque) by saturating *e* and prevents the plant from peaking. It contains both linear and nonlinear gains. The high-gain observer block implements Eq 28 and estimates the delayed states. It takes saturated control input from controller and reduces the observer dynamics to Eq 30 by canceling the system’s nonlinearities. The feedforward and feedback torque complete the loop to the nonlinear system. The whole simulation scheme mimics the CNS to ensure stable and physiologically relevant body movement during STS transfer. This scheme offers an interpretation of CNS emulation, in the presence of delays, to meet proper STS physiological movement dynamics.

**Fig 3:**
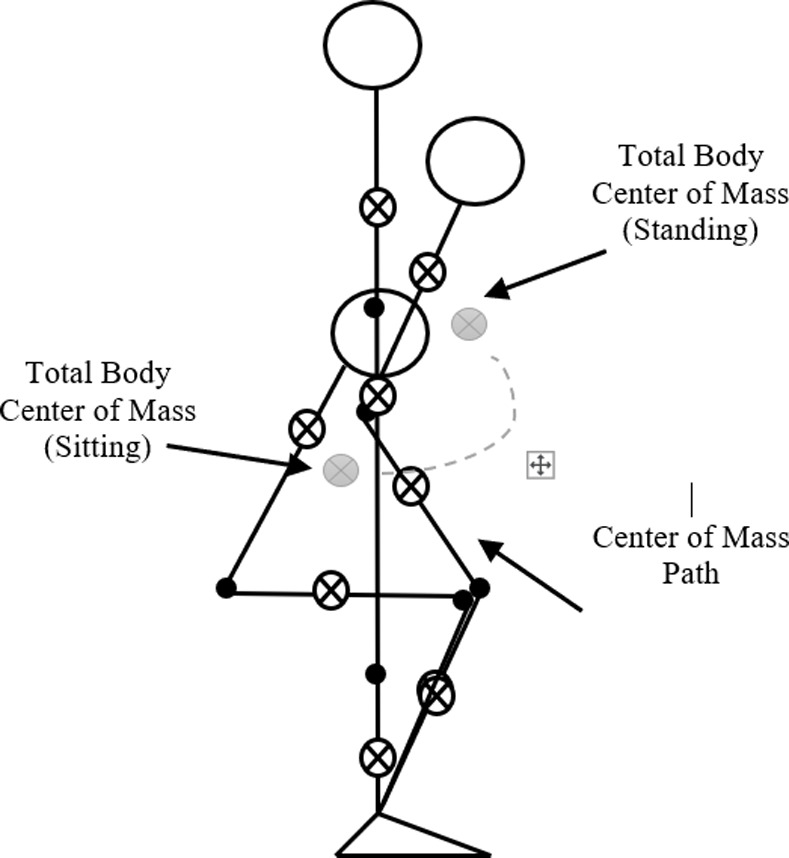
Sit-to-stand transition. Center-of-mass, head, ankle, knee, and hip joint movement during STS transition in a simple biomechanical model. The initial position is sitting, and the final position is standing.

The initial $\left[\frac{\pi }{2},0,\frac{\pi }{2}\right]$ rad and terminal $\left[\frac{\pi }{2},\frac{\pi }{2},\frac{\pi }{2}\right]$ rad position during STS transfer are shown in Fig 3. We obtain the simulation results for the joint position, velocity, torque, COM, GRF, and head position. All simulations are done in SI units i.e., time in seconds (s), force in Newtons (N), torque in N-m, and angles in radians (rad). Simulation results show the comparison of profiles simulated with and without delays. We assume the initial angular velocity to be zero at the movement inception. Additionally, the lifting off the chair is presumed to have happened beforehand, and no chair and/or hand reaction forces are considered.

**Fig 4:**
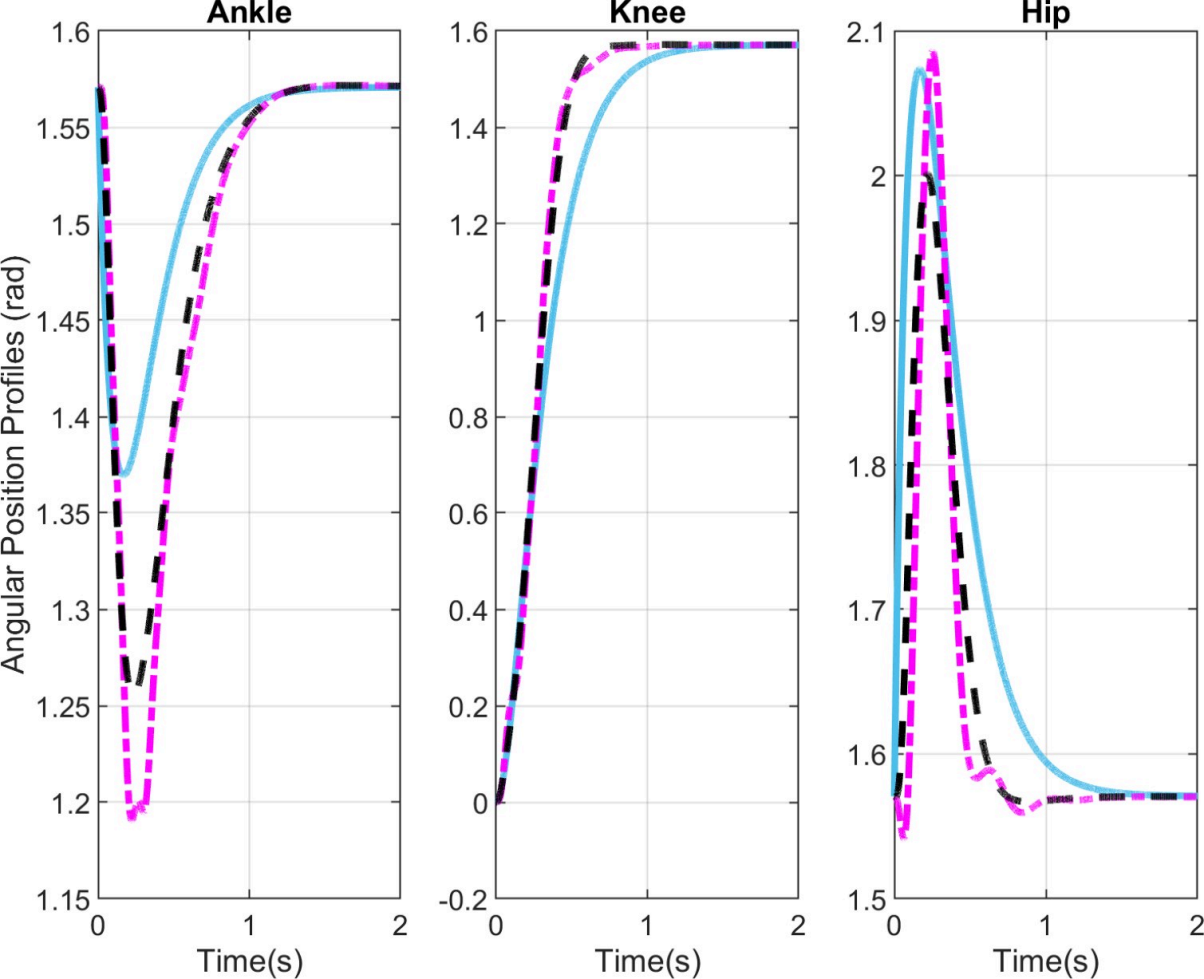
Angular position profiles. Blue lines show the reference position profiles, black lines show the simulation results without delays, and purple lines show the simulation results with delays.

Fig 4 shows the simulation results for the reference joint profiles and simulated joint position profiles with and without delays. These profiles are plotted in the ground frame of reference. The angular profiles show the nonlinear compensator tracks the reference trajectories and achieve the movement termination in 1.5s, which is the average time for STS transfer. The ankle and hip position profiles start and end at $\frac{\pi }{2}$ rad, whereas the knee joint profile starts at 0 rad and ends at $\frac{\pi }{2}$ rad. The ankle, knee, and hip joint profiles with and without delays are in phase with reference profiles, track the references smoothly without oscillations, and do not violate the physiological bounds. In previous studies by Rasool G et al. [21,23], the ankle and knee joint profiles go beyond 1.57 radians. Furthermore, the hip joint angle reduces to 1.45 rad during the extension phase with fuzzy LQR and is completely out of phase in case of *H*_2_ compensator design. In a recent study by Mughal et al. [24] the mixed *H*_2_/*H*_∞_ scheme shows an improvement over the earlier studies yet the ankle joint profile shows undesired deviations, oscillations, and higher dips. Moreover, one can observe the knee joint movement in opposite direction at the movement initiation.

**Fig 5:**
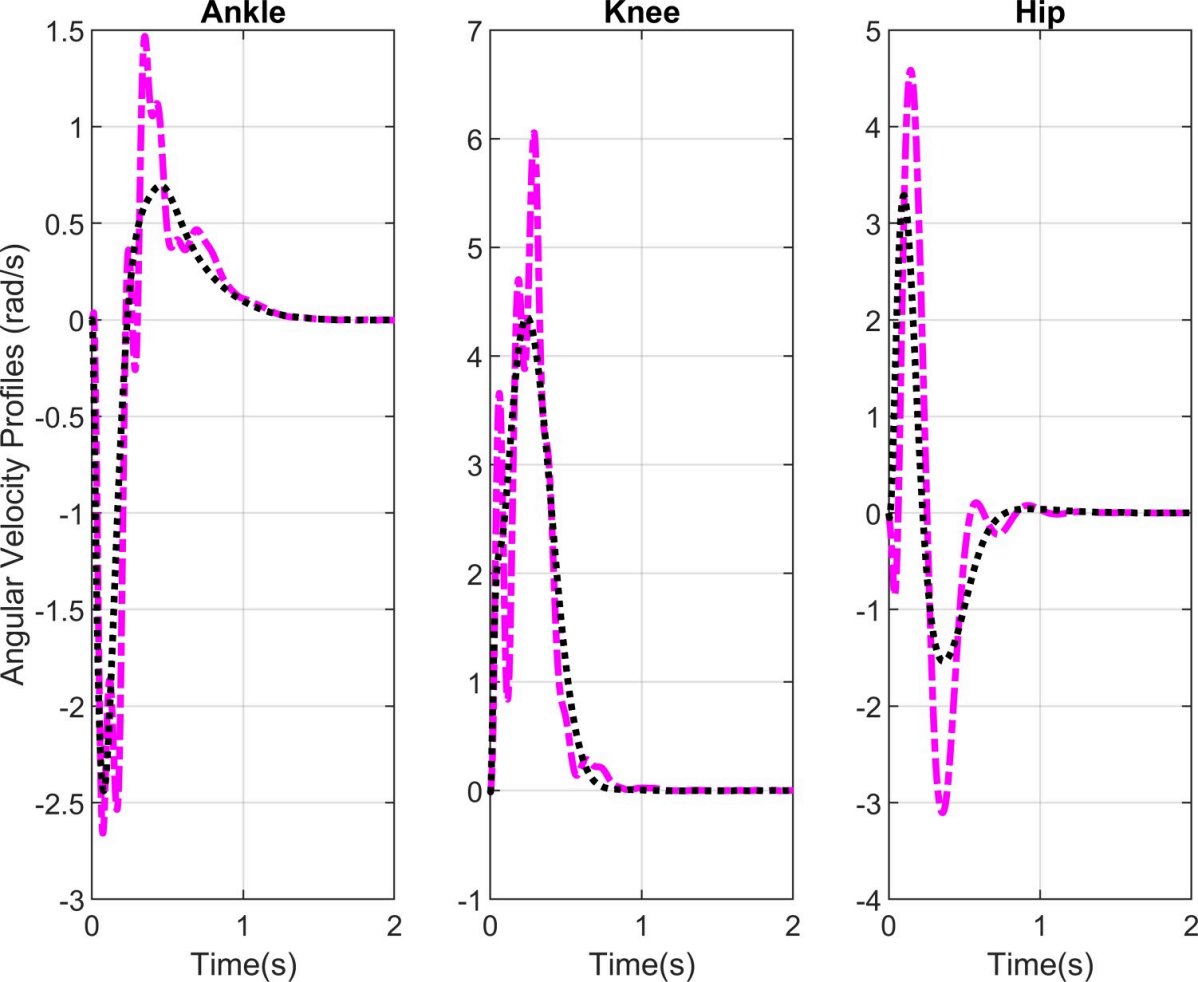
Angular velocity profiles. Black lines show the simulation results without delays and purple lines show the simulation results with delays.

The angular velocity profiles in Fig 5 shows that the movement terminates in 1.5s with and without delays. The profiles show a bit higher overshoots in case of delays. The initial angular velocity is zero since we are not taking into consideration the seat reaction forces. The desired final angular velocity is 0 rad. Physiologically the movement terminates when the hip velocity reaches zero.

**Fig 6:**
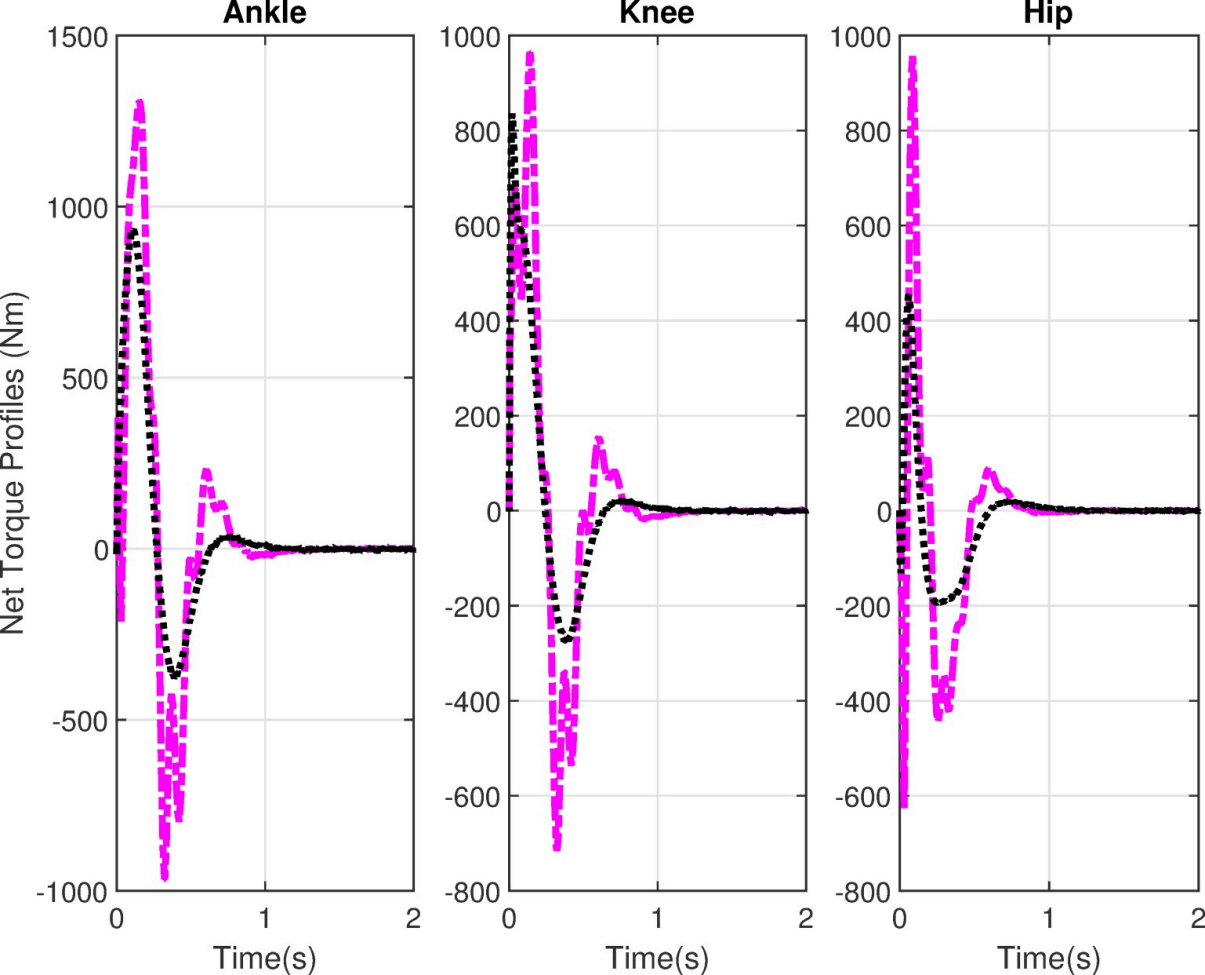
Joint torque profiles. Black lines show the simulations without delays and purple lines show the simulations with delays.

**Fig 7:**
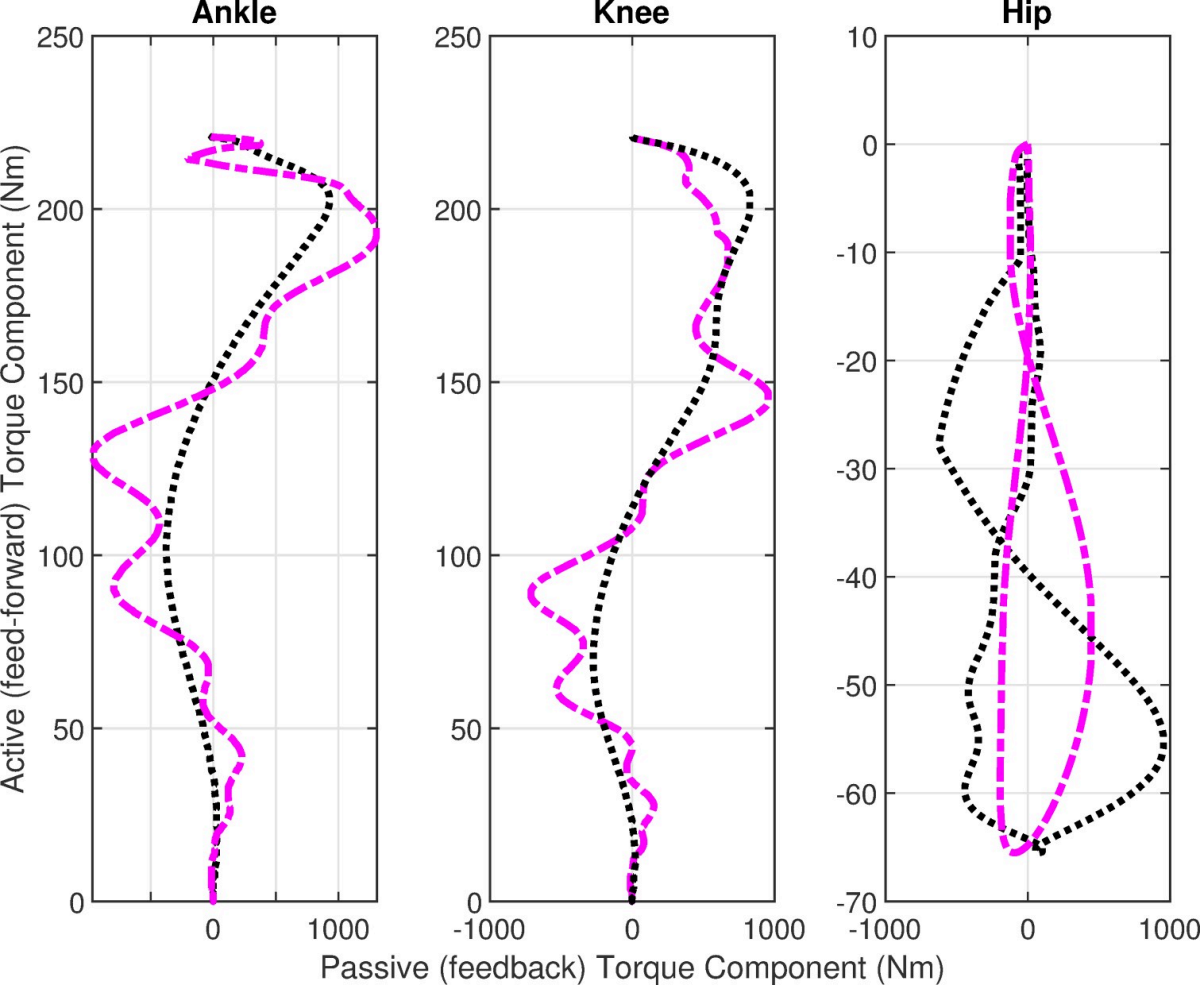
Active vs passive profiles. Black lines show the simulations without delays and purple lines show the simulations with delays.

Fig 6 shows the net joint torque profiles. The joint torque shows higher values in case of delays, which indicates that more effort is required in the successful execution of STS movement with feedback delays. These profiles are recast into Fig 7 which shows the comparison of active, and passive torques. The active or feedforward torques are assumed by CNS for the execution of STS movement. The feedback torques show passive viscoelasticity at joints and are higher than the active torques. The torque profiles show an initial flexion followed by extensor torque which facilitates the upward motion during the extension phase. The torque profiles settle down in 1.2s for both cases.

**Fig 8:**
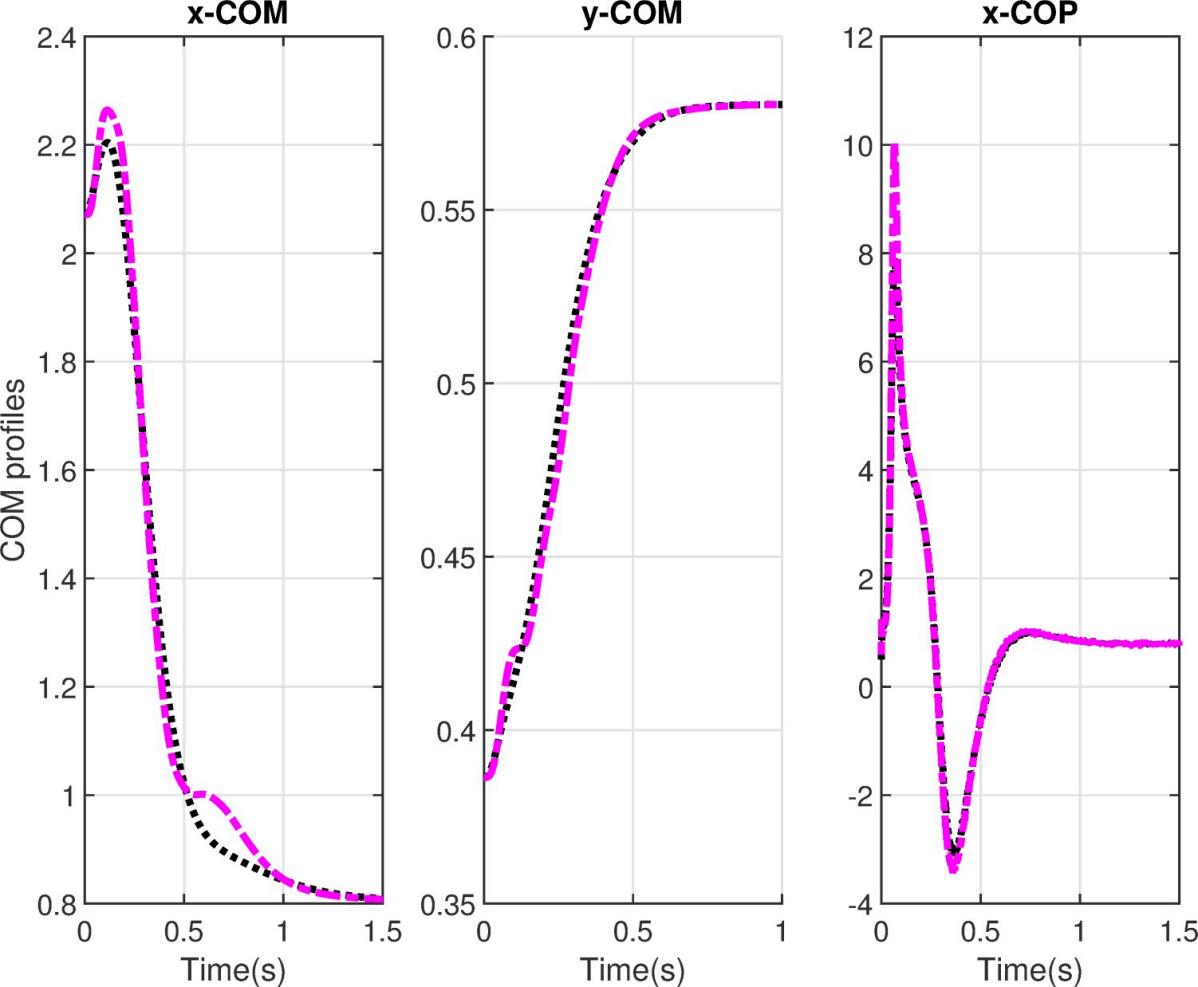
Ground reaction forces profiles. The horizontal GRF-x and vertical GRF-y with and without delays. Black lines show the simulations without delays and purple lines show simulations with delays.

Fig 8 shows the simulation results for physiological variables GRF-x and GRF-y. The horizontal GRF component settles to zero in 1.2s, whereas the vertical GRF component settles at bodyweight 650N in less than 1s. The initial peak shows the forward thrust of the body; the other peaks are due to limb movements which take time in settling down. The GRF-x takes a longer time in settling because of the body balancing after motion. The GRF profiles show an overshoot in case of delays. The GRF-y profiles match the GRF profiles in the vertical direction of the experimental GRF profiles [44] in terms of shape for both with and without delay cases. The GRF-y profile with delays shows much higher peaks in amplitude which indicates more forward thrust and limb movements before reaching a steady state. These profiles show that the simulated postural movement is physiologically relevant to the experimental data.

**Fig 9:**
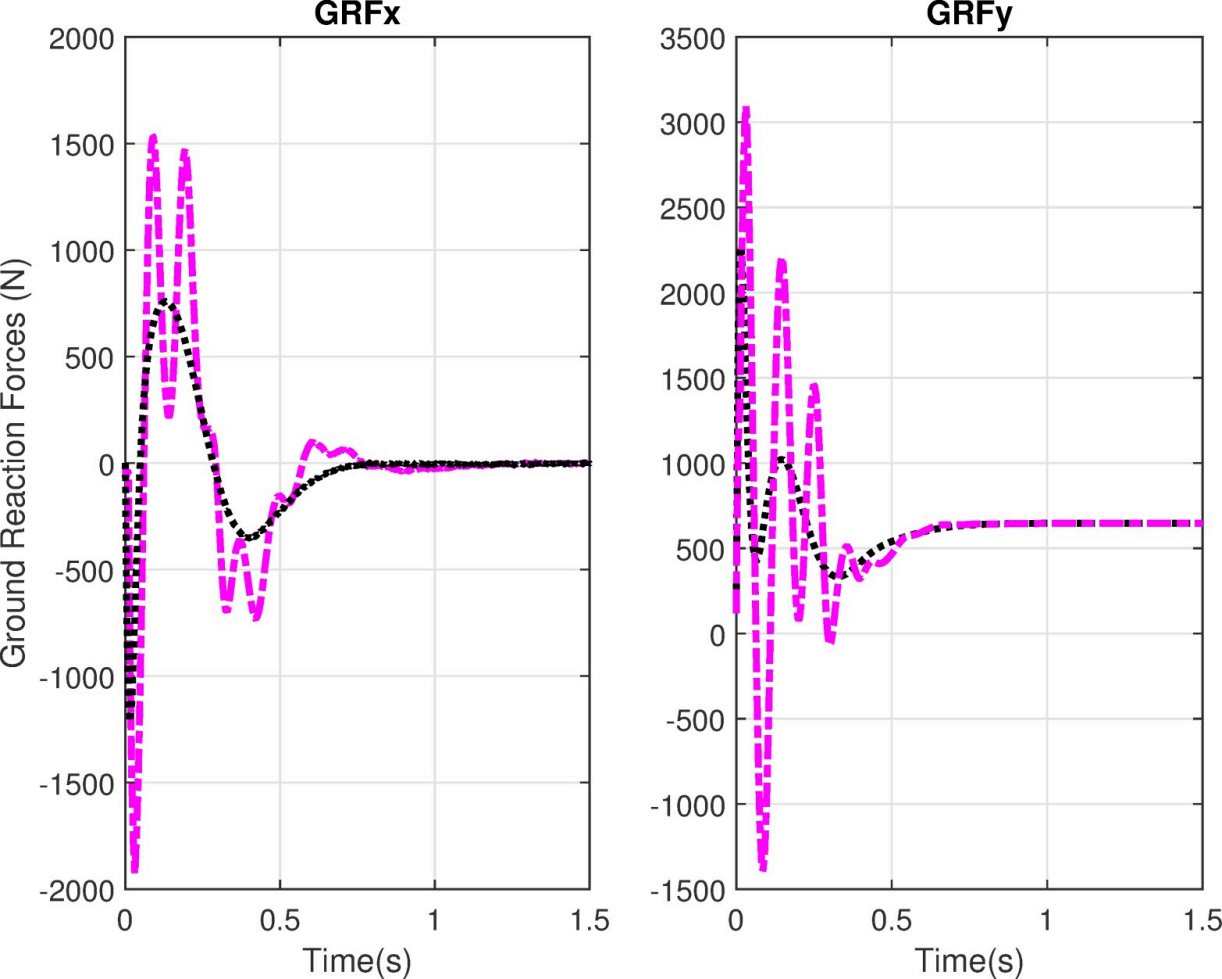
Center-of-mass and center-of-pressure profiles. The normalized horizontal x-COM, vertical y-COM, and horizontal x-COP components. Black lines show the simulations without delays and purple lines show the simulations with delays.

**Fig 10:**
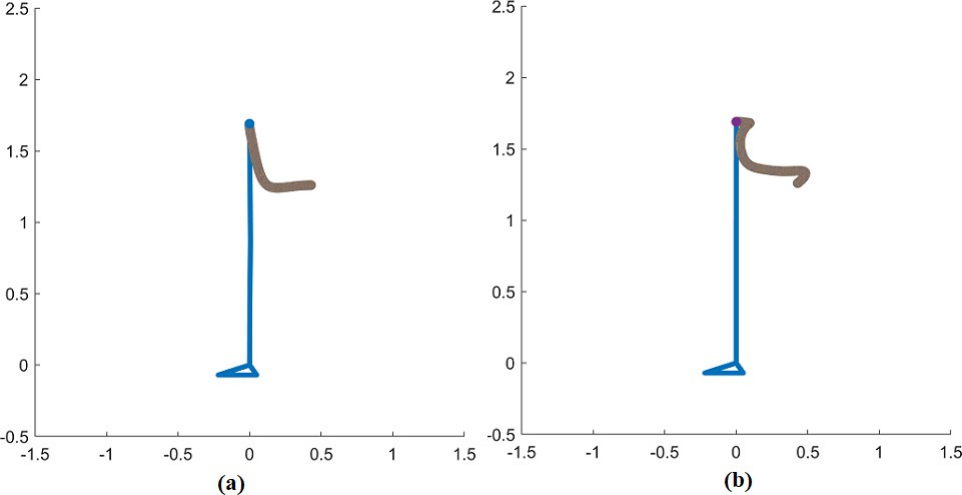
Head potion trajectory a) Reference head trajectory b) Head trajectory motion with delays.

We present the simulation results for the physiological variable COM and COP in Fig 9. The horizontal COM and COP component x-COM, x-COP are normalized at the foot length *l*_*f*_ and show smooth response for both profiles with and without delays. The profiles get settled in 1.5s at 80% of the foot length which shows a stable upright posture. The vertical COM components y-COM is normalized at the body height (*b* + *l*_1_ + *l*_2_ + *l*_3_) and show smooth COM movement for simulations with and without delay. The profile settles at 58% of the body height.

(Fig 10A and 10B) shows the reference head trajectory based on experimental data [39] and the head trajectory motion profile with delays. The simulated head trajectory profile during STS movement closely matches in shape to the reference head trajectory. The simulated head trajectory profiles show a physiologically relevant and correct STS movement.

We present in Table 3 the comparison of our work with the earlier work done with feedback delays. We compared different aspects of STS position profiles. It is desirable for physiologically correct STS movement that the ankle joint angle stays within 1.57 rad. The initial ankle angle during sitting position is 1.57 rad with respect to ground. When STS movement starts the ankle angle reduces during the forward thrust phase and increases again during the upward extension phase. At the STS movement termination, the ankle angle is 1.57 rad. Our simulation results for the ankle joint show physiologically correct STS movement. The ankle movement in opposite direction shows that the profiles cross the physiological boundaries as in the case of the study conducted by Rasool et al. [21,23], the ankle joint shows movement in opposite direction i.e. beyond 1.57 rad. This is due to linearization. Rasool et al. [21,23] conducted a study on STS movement using 4-segments biomechanical model in the presence of proprioceptive feedback delays. They linearized the model at sitting and standing position and approximated the delays with 2^nd^ order Pade approximation. This scheme increased the 6^th^ order nonlinear system to two 18^th^ order linear systems. Moreover [21,23,24] show a higher dip in the ankle angle as compared to the reference ankle angle. Our simulations show a max dip in ankle angle 0.37 rad which is close to the reference ankle angle. The knee joint angle cannot exceed 1.57 rad due to physiological constraints. The knee joint profiles [21] show an increase in angle up to 1.7 rad illustrating 0.13 rad beyond the physiological limit. The hip joint profile is completely out of phase with respect to the reference profile [23]. These problems were solved with LMI mixed *H*_2_/*H*_∞_ scheme [24] but at the cost of other physiological aspects. The maximum dip in ankle angle is still high. Also, one can observe oscillations in the profile. The knee joint profile with a mixed *H*_2_/*H*_∞_ scheme shows an initial knee movement in the opposite direction. The authors used a linearized model in their simulation scheme and approximated delays with Pade approximation which increased the system model from 6^th^ to 18^th^ order. Moreover, the ankle, knee, and hip joint position profiles show a high correlation of 0.91, 0.97, 0.80 respectively with the reference profiles. Our simulation scheme takes into account the system’s nonlinearities and compensates proprioceptive feedback delays and noise in joint sensors without increasing the system’s order.

**Table 3:** Comparison of our simulated STS movement with earlier work in the presence of proprioceptive feedback delays.

Position profiles with feedback delays (rad)	Ref Trajectories	Proposed framework	Ref [21] Fuzzy LQR	Ref [23] *H*_2_	Ref [24] LMI mixed *H*_2_/*H*_∞_
Ankle movement in opposite direction (beyond 1.57 rad)	0	0	0.28	0.23	0
Maximum dip in Ankle angle (rad)	0.22	0.37	0.47	0.87	0.67
Knee movement in opposite direction (beyond 1.57 rad)	0	0	0.13	0	0
Maximum dip in knee angle (rad)	0	0	0	0	0.4
Hip movement in opposite direction (beyond 1.57 rad)	0	0	0.12	0.57 (out of phase)	0
The maximum rise in hip angle (rad)	0.52	0.53	0.18	NA	0.54
Settling time (s)	1.5	1.5	4	4	2.5

## 5. Discussion and conclusion

In our current study, we investigated the design of a robust nonlinear compensator comprising of feedback linearization controller and high-gain observer, for simulations of STS movement in the presence of physiological feedback delays and noisy joint sensors. The model studied in this paper is based on several simplifying assumptions. We did not explicitly model the afferent feedback from MS, GTO, and spinal reflexes. The musculotendon dynamics are modeled as the time delay between the motor command and feedback torque generation and the study is limited to the sagittal plane only.

In the proposed simulation scheme, we calculated the linear gains of the controller and observer with the *H*_2_ compensator design. These gains were optimized with the sensitivity derivative of physiological variables COM and GRF. The relative contribution of active control (feed-forward) and passive stiffness (feedback) was also analyzed. The model used in this study is comprised of 4-segments representing feet, legs, thighs, and HAT (head-arm-trunk) in the sagittal plane mechanics. The torque at each joint includes the active component which represents the muscle stimulation in response to active commands from CNS and the feedback component which represents the passive viscoelasticity of joints. Thus, the torque actuation at the ankle, knee, and hip joint mimics the biological muscle function in performing the STS movement. The model includes the proprioceptive (MS and GTO) delays in the feedback path however, we did not explicitly model the afferent feedback from MS and GTO. The feedback delays are included in the ankle, knee, and hip joint position and velocity. The reference trajectories for STS movement were generated from a third-order linear model based on experimental data [39]. The respective derivatives of reference angles were calculated from the third-order model and no derivative blocks were used in Simulink for the calculation of derivatives. The feedback linearization control law under state feedback is represented in Eq 15. To estimate the unobserved delayed states, we need the control law with output feedback. We designed a high-gain observer to estimate the delayed states due to its properties of robustness against uncertainties and large perturbations. One important feature of the high-gain observer is its performance recovery i.e., when the states are provided by the high-gain observer under a sufficiently small value of *ε*, the output feedback controller can provide very similar performance to the state feedback case. But the problem of peaking arises due to the high gain i.e., there will be an initial sharp spike in the response of the state estimates. Which can make the system unstable. This problem is solved by saturating the controller outside the operating range of the system states as shown in Eqs 29 and 30. The position profiles simulation results reflect the performance of high-gain observer-based control. This is due to the combined effect of the global boundness of the controller and fast observer dynamics. The passive torques at joints are zero at the movement initiation and they start building up as the movement progresses, it is then followed by recession and finally settling at zero. The delays affect the passive torques significantly and one can observe the increase in torque magnitude for smooth, stable, and timely execution of STS movement. The head trajectory profile resembles in shape the experimental head trajectory profile of the study reported by Samina et al. [4], validating the physiological relevance of the simulation scheme. The STS movement is a complex neurophysiological process, and, in this study, we presented a simulation scheme based on a nonlinear compensator design to mimic the complex behavior of CNS in controlling STS movement in the presence of neural delays. These physiological delays have major effects on the performance of the STS movement in elderly and neuro deficient persons. Researchers used different techniques e.g. hyperbolic functions [45], Bessel functions [46], Laguerre polynomials [47] and Pade approximation [21,23,24] to approximate delays. The simulation results of our study show an improvement over previous studies in terms of transient responses and settling time and support our hypothesis of considering the system’s nonlinearities and nonlinear control scheme for better emulation of CNS in controlling STS movement.

The nonlinear simulation scheme in combination with *H*_2_ provides a new prospect to this research. This study is useful in the diagnosis of STS movement coordination abnormalities related to kinematic senses. They can further be quantified, and rehabilitated, thus beneficial in rehabilitation robotics as well. We aim to extend this study to explore the role of the extended high-gain observer in controlling the STS movement of 2D and 3D bipedal models for better insight into STS motion of healthy and neuro-deficient subjects. This technique can be used further to elaborate on the stroke patient’s asymmetric motion profiles and research the postural stability to prevent elderly fall prevention.

## Appendix A


$$C_{y}\left[\begin{array}{cccccc} 267390 & 0 & 0 & 0 & 0 & 0\\ 0 & 250990 & 0 & 0 & 0 & 0\\ 0 & 0 & 711050 & 0 & 0 & 0\\ 0 & 0 & 0 & 45960 & 0 & 0\\ 0 & 0 & 0 & 0 & 41250 & 0\\ 0 & 0 & 0 & 0 & 0 & 89620 \end{array}\right]$$

$$D_{yu}=\left[\begin{array}{ccc} 2.3045 & 0 & 0\\ 0 & 4.8403 & 0\\ 0 & 0 & 2.7697 \end{array}\right]$$

$$A-BK=\left[\begin{array}{cccccc} 0 & 0 & 0 & 1 & 0 & 0\\ 0 & 0 & 0 & 0 & 1 & 0\\ 0 & 0 & 0 & 0 & 0 & 1\\ -112640 & -9950 & 71220 & -19330 & -1740 & 8800\\ -800 & -47370 & -60840 & -230 & -7800 & -7790\\ -23890 & 36530 & -226240 & -4110 & 5940 & -28570 \end{array}\right]$$

$$P_{1}=\left[\begin{array}{cccccc} 0.0859 & 0.0007 & 0.0004 & -0.5000 & 0.0008 & -0.0023\\ 0.0007 & 0.0823 & 0.0005 & -0.0008 & -0.5000 & -0.0031\\ 0.0004 & 0.0005 & 0.0632 & 0.0023 & 0.0031 & -0.5000\\ -0.5000 & -0.0008 & 0.0023 & 2.9133 & -0.0230 & -0.0246\\ 0.0008 & -0.5000 & 0.0031 & -0.0230 & 3.0421 & -0.0289\\ -0.0023 & -0.0031 & -0.5000 & -0.0246 & -0.0289 & 3.9549 \end{array}\right]$$

Closed loop eigen value = [−550.7 −21+21*i* −21−21*i* −12 −5+5*i* −5−5*i*]
